# Dr. Annie M. Tremaine

**Published:** 1912-04

**Authors:** 


Dr. Annie M. Tremaine of Fredonia, died at Willard, N. Y., March 25, 1912.

